# The natural variance of Arabidopsis secondary metabolism on extended darkness

**DOI:** 10.1038/s41597-024-03694-2

**Published:** 2024-08-03

**Authors:** Feng Zhu, Micha Wijesingha Ahchige, Weiwei Wen, Yunjiang Cheng, Saleh Alseekh, Alisdair R. Fernie

**Affiliations:** 1 National Key Laboratory for Germplasm Innovation & Utilization of Horticultural Crops, National R&D Center for Citrus Preservation, Huazhong Agricultural University, 430070 Wuhan, China; 2 Hubei Hongshan Laboratory, 430070 Wuhan, China; 3 Max-Planck-Institut für Molekulare Pflanzenphysiologie, Am Mühlenberg 1, 14476 Potsdam-Golm, Germany; 4 Center of Plant Systems Biology and Biotechnology, 4000 Plovdiv, Bulgaria

**Keywords:** Secondary metabolism, Natural variation in plants

## Abstract

In plants due to their sessile nature, secondary metabolites are important components against different abiotic and biotic stress, such as extended darkness. For this reason, the variation of secondary metabolite content of the *Arabidopsis thaliana* HapMap natural population following 0-and 6-d darkness treatment were detected and the raw data of different accessions at two timepoints were deposited in the Zenodo database. Moreover, the annotated secondary metabolites of these samples are presented in this data descriptor, which we believe will be a usefully re-usable resource for future integrative analysis with dark-treated transcripts, proteins or other phenotypic data in order to comprehensively illustrate the multiomic landscape of Arabidopsis in response to the stresses exerted by extended darkness.

## Background & Summary

Being significantly different to primary metabolites which are the simple structure components that harbor vital roles in development, plant secondary metabolites are derived from the primary metabolites and non-essential for the normal metabolism of the organism but exhibited the survival function different abiotic and biotic stresses^1–4^. For example, although sunlight is the energy resource of plant, ultraviolet (UV) light whose wavelength ranges from 280 to 320 nm (UV-B) potentially causes the damage of nucleic acid and proteins and promotes transposon activity which may induce mutations^5^. Our former study has demonstrated that the content of phenylacylated-flavonols (saiginols) is high-correlated with UV light intensity and the production of saiginols is an important mechanism of Arabidopsis against the UV light damage^1,6^. Unlike the damage of UV radiation on nucleic acid and proteins, the extended darkness stress causes the cessation of photosynthesis and nutrient starvation of plant. The genomic landscape of primary metabolite and lipids have been illustrated but the genetic regulation of secondary metabolites under extended darkness stress is still unknown^7^. In general, secondary metabolites have been divided into different classification based on the characteristic chemical structures, such as phenolic acids, flavonoids, terpenoids and steroids, and alkaloids^8^. Recently, with the explosive development of mass spectrometry methods, liquid chromatography-mass spectrometry (LC-MS) has widely been used as the high-throughput method to analyze the complex plant secondary metabolism^7,9–11^.

**Fig. 1:**
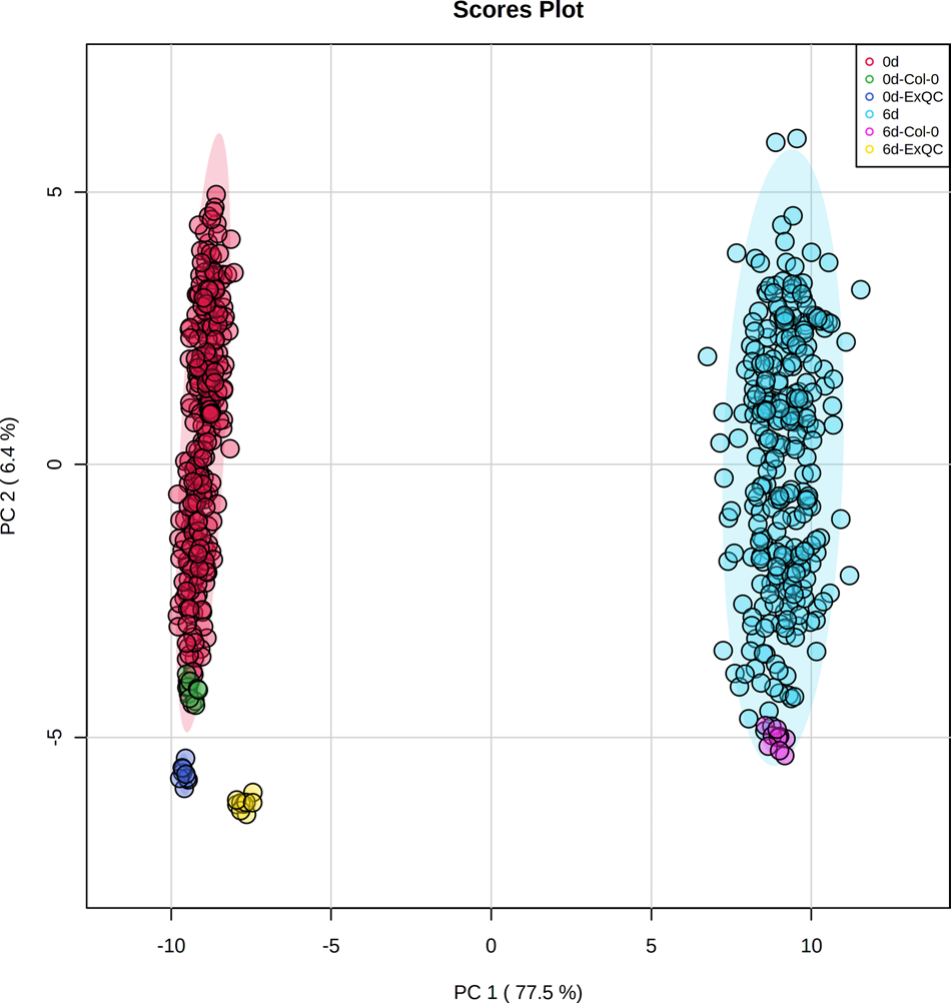
The principle component analysis of metabolite levels of different Arabidopsis accessions for two time points.

**Fig. 2:**
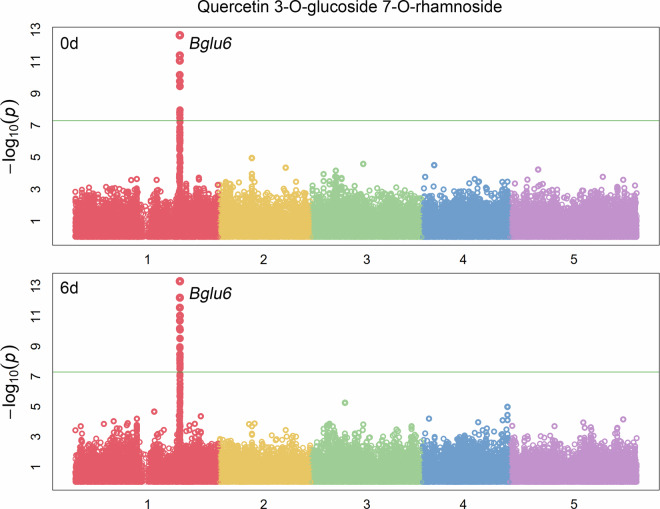
The Manhattan plot of the association of Bglu6 (AT1G60270) and Quercetin 3-O-glucoside 7-O-rhamnoside under 0d and 6d after darkness treatment. The colour and numbers of the x axis were indicated the different chromosomes of Arabidopsis genome.

**Fig. 3:**
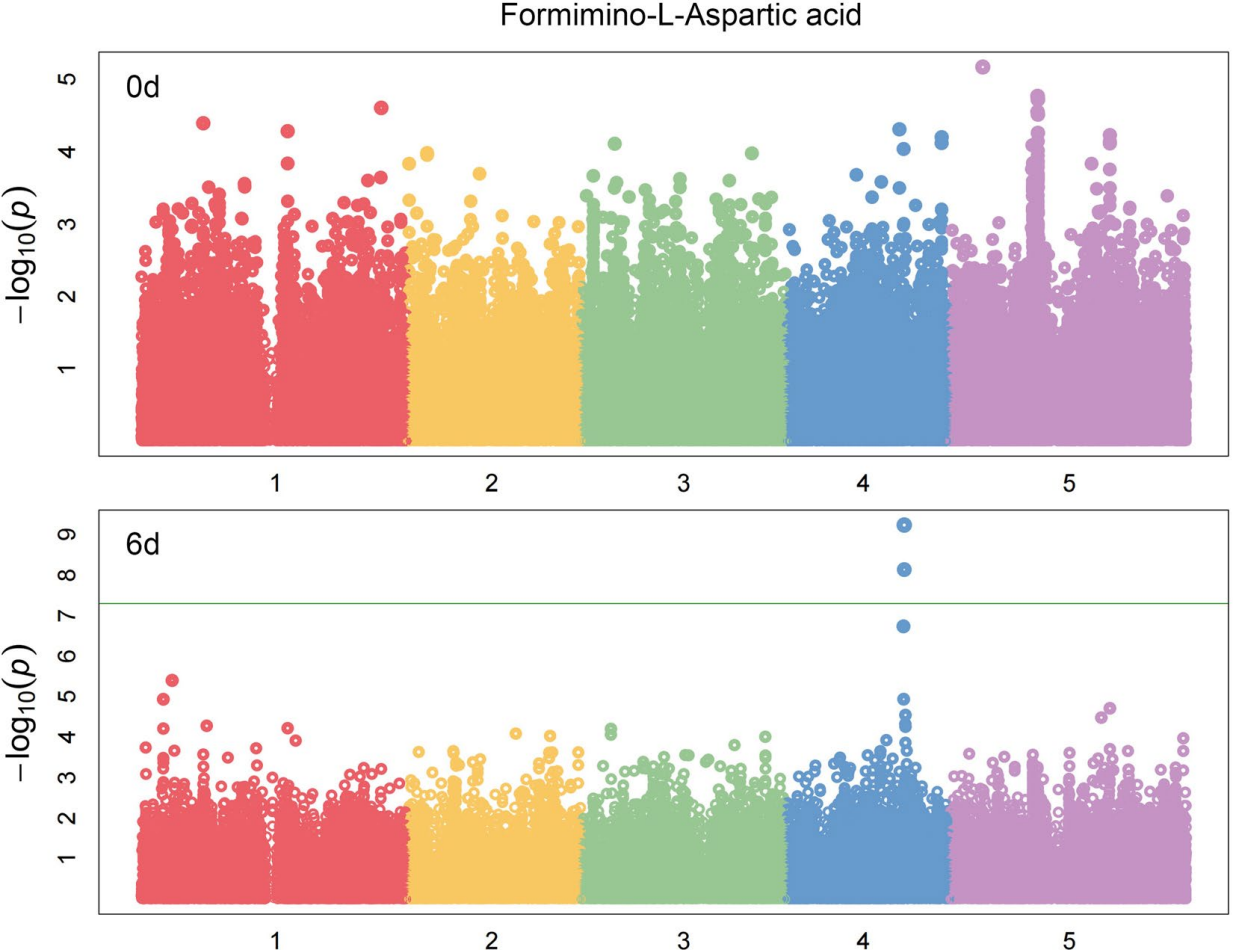
The Manhattan plot of the association of formimino-L-Aspartic acid under 0d and 6d after darkness treatment. The colour and numbers of the x axis were indicated the different chromosomes of Arabidopsis genome.

Here, the raw data of secondary metabolites of *Arabidopsis thaliana* HapMap natural population after 0d and 6d darkness treatment are deposited to zenodo (https://zenodo.org/)^12–24^. Among the samples, 95 metabolites were well-annotated based on standards, or MS/MS data from former studies^1,25–27^ and the detailed information of the metabolites content of different accession in different timepoints can been found in figshare^28,29^. A principal component analysis (PCA) of the different time points and accessions based on the secondary metabolites content exhibited the significantly separation between 0d and the 6d samples, indicating the remarkably effect of darkness on the secondary metabolome (Fig. 1)^30^. Genome-wide association study (GWAS) analysis based on the data in our present study identified the strong associations between the well-known gene (*Bglu6*, AT1G60270) with Quercetin 3-*O*-glucoside 7*-O*-rhamnoside, which indicated the high quality of the data normalization pipeline and the accuracy of the analysis concerning the different metabolite intensities between time points (Fig. 2)^31^. Moreover, some novel associations such as with formimino-L-aspartic acid which can just be identified in 6d after dark-treated dataset (Fig. 3)^32^ indicated that extend darkness can significantly change the genetic regulation of metabolites and further supported that these data could be used not only in future to further mine the novel associations with secondary metabolites but also in the mutiomics analysis alongside dark-treated transcriptome, proteome and phenome data in order to figure out the global regulation of Arabidopsis against the extended darkness stress.

## Methods

### Plant material and sample preparation

The seeds of 259 Arabidopsis accession of *Arabidopsis thaliana* HapMap natural population were grown on soil under a short-day (SD) photoperiod in a greenhouse for 5 weeks. At 35-days after germination, one plant of each accession was harvested (0 days) and quickly frozen in liquid nitrogen and after 6d,another plant of each accession was harvested within 30 min at 10 am (GMT + 2). All samples were stored at –80 °C prior to further analysis. The positioning of the plants was randomized to avoid block effects during growth and one Col-0 plant was grown in each tray to analyze the spatial variation between different trays. Moreover, the experiment was performed twice in autumn 2018 and spring 2019 as two independent biological replicates. Two pattern quality controls were included in the experiment to remove extraction and batch effects for the final processed data. First, one Ex-QC sample from the same pool material before extended darkness treatment was added per 40 analyzed samples during metabolite extraction and followed the same analytical pathway. Second, an identical quality control was added to every 14 samples during metabolite profiling, and each batch of 60 samples (including four quality controls) was then standardized to the four quality controls^7^.

### LC-MS analysis

The extraction of metabolites was carried out following the method described in a previous study^33^. One mL pre-cooled extraction solvent (methyl tert-butyl ether/methanol 3:1 vol/vol) was added to 50 mg grinded Arabidopsis leaf. The tube containing sample and extraction buffer was vortexed for 1 min and then shaken on an orbital shaker (100 rpm) for 45 min at 4 °C and sonicated for 15 min. An additional 500 µL of phase separation buffer (water/methanol 3:1 vol/vol) was added to tube and the samples were thoroughly vortexed again for 1 min. After that, the samples are centrifuged at a speed of 20,000 g for 5 min at 4 °C. The 360 µL polar phase underwent drying using a SpeedVac concentrator and was then suspended in 150 µL of 50% methanol (methanol/water 50:50 vol/vol). Then, 3 μL of supernatant was injected and analyzed by the Waters Acquity UPLC system coupled to an Exactive Orbitrap mass detector according to the previously published protocol^33^. 0.1% formic acid in water (Solvent A) and 0.1% formic acid in acetonitrile (Solvent B) were used as mobile phases. The mobile phase flow rate was 400 µL/min. The spectra were captured in negative ion detection full scan mode, covering a mass range of 100 to 1500 m/z. The maximum scan time was set to 250 ms and the resolution was set to 25,000. From 0 min to min 19 of the UPLC gradient, MS spectra were recorded. RefinerMS software (version 5.3; GeneData), MetAlign^34^, and Xcalibur software files (Thermo Fisher Scientific) were used to extract molecular masses, retention times, and corresponding peak intensities from the raw. Metabolite identification and annotation were performed using in house standard library, MS^2^ fragmentation battens, literature base, and metabolomics databases^1,9,25,27^. Metabolites are reported following the recently updated reporting standards for metabolomics^9^.

### Data processing and metabolite data analysis

The data was analyzed with the Xcalibur 2.1.0 program, and peak identification and annotation were carried out by integrative method: standard chemical confirmation, MS fragmentation and retention time profiling^1,25,27,35^. Xcalibur Quan Browser was used to choose the best peaks under the parameter as Window (sec), 30; highest peak; minimum peak height (S/N), 3.0; Baseline window, 80–150; area noise factor, 2; peak noise factor, 10; peak height (%), 5.0, tailing factor, 1.5^1,25,27,35^.

### GWAS analysis

The normalized metabolite intensity values were used to map the phenotypic observations to loci in the *A. thaliana* genome based on 199455 SNP markers (minor allele frequency > 1%) of Affymetrix GeneChip Array 6.0^36,37^. The SNP fraction parameter was set to 1, and other parameters were set to default values as recommended by the GAPIT user manual. The genome-wide suggestive and significant threshold of SNP was set to *p*-value = 3.16 × 10^−5^ and 5.01 × 10^−6^, respectively as described previously^38^.

## Data Records

A total of 1244 raw data of the different conditions and Arabidopsis accessions and quality control samples of two different years have been deposited to zenodo (https://zenodo.org/)^12–24^. The detailed information of the annotated metabolites and metabolites content of different accessions in different timepoints were deposited in the figshare (10.6084/m9.figshare.24407896.v3 and 10.6084/m9.figshare.24407812.v3)^28,29^.

## Technical Validation

Two pattern quality controls have been included in the analysis to ensure the accuracy of the analysis about the different metabolite intensity between different conditions. The metabolites result of two different years’ samples have been used to calculate the best linear unbiased predictions (BLUPs) using the R package *lme4*^39^.

## Usage Notes

Figure 1 shows the principle component analysis of metabolite levels of different Arabidopsis accessions for two time points^30^. Figure 2 shows the Manhattan plot of the association of *Bglu6* (AT1G60270) and Quercetin 3-*O*-glucoside 7-*O-*rhamnoside under 0d and 6d after darkness treatment^31^. Figure 3 shows the Manhattan plot of the association of formimino-L-Aspartic acid under 0d and 6d after darkness treatment^32^.

## Data Availability

Genome Association and Prediction Integrated Tool (GAPIT) R package: https://www.maizegenetics.net/gapit^40^. The principle component analysis were performed using MetaboAnalyst 6.0^41^.
